# Imbalanced Inflammatory Responses in Preterm and Term Cord Blood Monocytes and Expansion of the CD14^+^CD16^+^ Subset upon Toll-like Receptor Stimulation

**DOI:** 10.3390/ijms24054919

**Published:** 2023-03-03

**Authors:** Kirsten Glaser, David Kern, Christian P. Speer, Nicolas Schlegel, Michael Schwab, Ulrich H. Thome, Christoph Härtel, Clyde J. Wright

**Affiliations:** 1 Center for Pediatric Research Leipzig, Division of Neonatology, Department of Women’s and Children’s Health, University of Leipzig Medical Center, 04103 Leipzig, Germany; 2 Department of Pediatrics, University Hospital Würzburg, 97080 Würzburg, Germany; 3 Department of General, Visceral, Transplantation, Vascular and Pediatric Surgery, University of Würzburg, 97080 Würzburg, Germany; 4 Department of Obstetrics and Gynecology, University of Würzburg, 97080 Würzburg, Germany; 5 Section of Neonatology, Department of Pediatrics, University of Colorado School of Medicine, Children’s Hospital Colorado, Aurora, CO 80045, USA

**Keywords:** neonatal immunology, inflammation, preterm infants, monocytes, cord blood, monocyte subsets, cytokines, Toll-like receptor signaling

## Abstract

Developmentally regulated features of innate immunity are thought to place preterm and term infants at risk of infection and inflammation-related morbidity. Underlying mechanisms are incompletely understood. Differences in monocyte function including toll-like receptor (TLR) expression and signaling have been discussed. Some studies point to generally impaired TLR signaling, others to differences in individual pathways. In the present study, we assessed mRNA and protein expression of pro- and anti-inflammatory cytokines in preterm and term cord blood (CB) monocytes compared with adult controls stimulated ex vivo with Pam3CSK4, zymosan, polyinosinic:polycytidylic acid, lipopolysaccharide, flagellin, and CpG oligonucleotide, which activate the TLR1/2, TLR2/6, TLR3, TLR4, TLR5, and TLR9 pathways, respectively. In parallel, frequencies of monocyte subsets, stimulus-driven TLR expression, and phosphorylation of TLR-associated signaling molecules were analyzed. Independent of stimulus, pro-inflammatory responses of term CB monocytes equaled adult controls. The same held true for preterm CB monocytes—except for lower IL-1β levels. In contrast, CB monocytes released lower amounts of anti-inflammatory IL-10 and IL-1ra, resulting in higher ratios of pro-inflammatory to anti-inflammatory cytokines. Phosphorylation of p65, p38, and ERK1/2 correlated with adult controls. However, stimulated CB samples stood out with higher frequencies of intermediate monocytes (CD14^+^CD16^+^). Both pro-inflammatory net effect and expansion of the intermediate subset were most pronounced upon stimulation with Pam3CSK4 (TLR1/2), zymosan (TR2/6), and lipopolysaccharide (TLR4). Our data demonstrate robust pro-inflammatory and yet attenuated anti-inflammatory responses in preterm and term CB monocytes, along with imbalanced cytokine ratios. Intermediate monocytes, a subset ascribed pro-inflammatory features, might participate in this inflammatory state.

## 1. Introduction

Advances in neonatal care have continuously improved the survival of preterm infants, yet morbidity remains high. Infection and inflammation-related morbidity are main risk factors for adverse outcomes. Observational and experimental studies indicate a heightened susceptibility of preterm and term neonates to imbalanced and sustained inflammation [1,2,3,4]—attributable, most likely, to unique features of innate and adaptive immune responses including monocyte capacities and toll-like receptor (TLR) signaling [4,5,6,7,8,9]. Underlying mechanisms are not yet fully understood.

Although accounting for only ~10% of nucleated blood cells, monocytes are key innate immune cells and critical effectors and regulators of inflammation [10]. Monocyte-derived pro-inflammatory tumor necrosis factor (TNF-) α, interleukin (IL-) 1β, IL-6, and IL-8 are among the first mediators to determine immediate host defense and prime adaptive immune responses [11,12]. Although formerly perceived as a homogenous population, there is increasing awareness of a considerable heterogeneity of circulating monocytes [13,14]. Based on CD14/CD16 surface expression, three potential subsets have been identified: classical (CD14^++^CD16^−^; ~80% of monocytes), intermediate (CD14^+^CD16^+^; about 2–10%), and non-classical monocytes (CD14^dim^CD16^+^; approx. 5–10%) [13,15]. In adults, the classical subset seems to be mainly phagocytic and a source of IL-6 and IL-8. Intermediate monocytes have been ascribed phagocytic and inflammatory function releasing TNF-α, IL-1β, IL-6, IL-8, and reactive oxygen species [13,14,16]. In addition, some studies have identified intermediate, and others classical, monocytes as the main source of IL-10 [16,17,18,19]. Non-classical monocytes appear to be poorly phagocytic and to release pro-inflammatory but no anti-inflammatory cytokines in response to viruses [16,18]. Alterations in subset frequencies have been associated with the onset and outcome of infectious, inflammatory, and cardiovascular diseases in adults [13,14,16]. In neonates, and especially in preterm neonates, far less is known about monocyte heterogeneity and its functional consequences [20].

Recognition of pathogens is mediated by pattern recognition receptors, with TLRs being the best characterized family [7,21]. Binding to cell surface TLRs (TLR1, 2, 4, 5, 6, 10) or TLRs localized to endosomal membranes (TLR3, 7, 8, 9) initiates specific signaling culminating in the release of inflammatory mediators [21]. Major signaling molecules include p65, a subunit forming the nuclear factor kappa-light-chain-enhancer of activated B cells (NF-κB) transcription factor, mitogen-activated protein kinase (MAPK) p38, extracellular signal-regulated kinase (ERK) 1/2, c-Jun N-terminal kinase (JNK), and interferon regulatory transcription factors (IRF) [21]. There is some evidence that individual signaling pathways might be impaired in neonates, while other pathways seem fully operable [22,23,24,25,26,27,28,29,30,31,32].

This study was designed to further investigate preterm and term cord blood (CB) monocyte capacities compared with the mature adult function and to explore the impact of monocyte subpopulations. We hypothesized that preterm and/or term CB monocytes would demonstrate variable responsiveness to individual TLR ligands, with some responses matching the adult response and others being immature and attenuated. Using RT-qPCR, cytokine flow cytometry, and bead-based multiplex assay, we assessed cytokine, chemokine, and interferon (IFN) responses (TNF-α, IL-1β, IL-6, IL-8, IL-12p40, IL-10, IL-1ra, CXCL10, CCL5, IFN-β, IFN-γ,) and TLR expression. Frequencies of monocyte subsets and phosphorylation of p65, p38, ERK1/2, JNK, IRF3, and IRF7 were analyzed by means of flow cytometry.

## 2. Results

### 2.1. Characteristics of Study Participants

Characteristics of CB donors are given in Table 1. All infants were born via caesarean section (C-section) to largely rule out confounding effects of potential antenatal and perinatal exposure to inflammation a priori. Postnatally, we excluded all CB donors with a history or clinical or laboratory evidence of chorioamnionitis (maternal fever > 38.0 °C, uterine tenderness, malodorous vaginal discharge, maternal leukocytosis > 15,000 cells/mL, maternal raised serum C-reactive protein > 5 mg/L, maternal tachycardia > 100 bpm and fetal tachycardia > 160 bpm) from our analysis as well as CB donors with suspected or confirmed perinatal infection (serum CRP > 5 mg/L and/or IL-6 > 100 pg/mL, tachycardia > 200 bpm, respiratory signs, lethargy, and fever > 38.0 °C or hypothermia). Additional placental examination did not find histopathological signs of chorioamnionitis in any CB donor. As far as adult donors were concerned, without exception, healthy adults were recruited. Finally, both neonatal and adult donors were excluded from our analyses if there was evidence of a “pre-activated inflammatory state” in unstimulated monocyte aliquots as determined by flow cytometry.

### 2.2. Robust Pro-Inflammatory Responses in TLR-Stimulated Preterm and Term Cord Blood Monocytes

To test whether early monocyte responses are selectively or globally impaired or fully operable in preterm and term neonates as compared to adults we assessed mRNA and protein expression of a broad variety of pro- and anti-inflammatory mediators in CB and adult monocytes exposed to Pam3CSK4, zymosan, polyinosinic:polycytidylic acid (poly(I:C)), lipopolysaccharide (LPS), flagellin, and CpG oligonucleotide (CpG ODN) which activate the TLR1/2, TLR2/6, TLR3, TLR4, TLR5, and TLR9 pathways, respectively—mimicking infection with gram-positive, gram-negative, and intracellular bacteria, viruses, and fungi. We analyzed pro-inflammatory cytokines that are broadly induced, such as TNF-α, IL-1β, IL-6, and IL-8, as well as mediators rather specific for individual pathways, such as CXCL10, CCL5, and IFNs, which are mainly released upon activation of TLR3 and TLR9.

**Figure 1:**
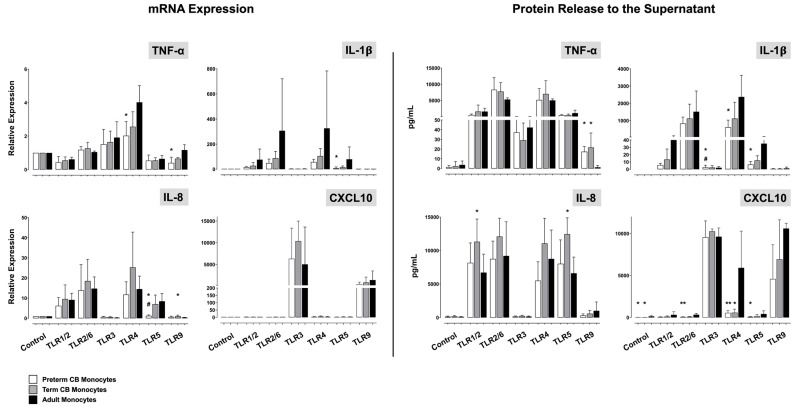
Pro-inflammatory TNF-α, IL-1β, IL-8, and CXCL10 responses in preterm CB (white bars), term CB (grey), and adult monocytes (black) upon stimulation with Pam3CSK4 (TLR1/2 ligand), zymosan (TLR2/6), transfected poly(I:C) (TLR3), LPS (TLR4), flagellin (TLR5), and CpG ODN (TLR9). Unstimulated monocytes served as controls (baseline expression). Relative quantification of cytokine mRNA and concentrations of secreted protein in the supernatant given in pg/mL are presented as mean ± SD (* *p* < 0.05, ** *p* < 0.01, versus adult monocytes; # *p* < 0.05, vs. term CB monocytes).

Independent of the stimulus used, term CB monocytes exhibited pro-inflammatory cytokine and chemokine responses comparable to adults both at the level of mRNA and protein expression (Figure 1, data presented for TNF-α, IL-1β, IL-8, and CXCL10). Upon stimulation with Pam3CSK4, poly(I:C), flagellin, and CpG ODN, of note, TNF-α and IL-8 release in term CB monocytes partly exceeded the adult release (Figure 1). Pro-inflammatory responses in preterm CB monocytes likewise equaled the adult response, except for IL-1β release, which was attenuated upon stimulation with poly(I:C), LPS, and flagellin compared with adults (Figure 1). As in term CB monocytes, CpG ODN-mediated TNF-α release was higher in preterm than in adult samples. Both preterm and term CB monocytes showed robust stimulus-driven expression of CXCL10 (Figure 1). IL-6 release was strongly induced upon stimulation with Pam3CSK4, zymosan, LPS, and flagellin, both in CB and adult monocytes. Stimulus-induced levels (mean 2.469–9.377 pg/mL) did not differ among study groups.

### 2.3. Attenuated Anti-Inflammatory Responses and Impaired IFN-γ Response in Neonatal Monocytes

**Figure 2:**
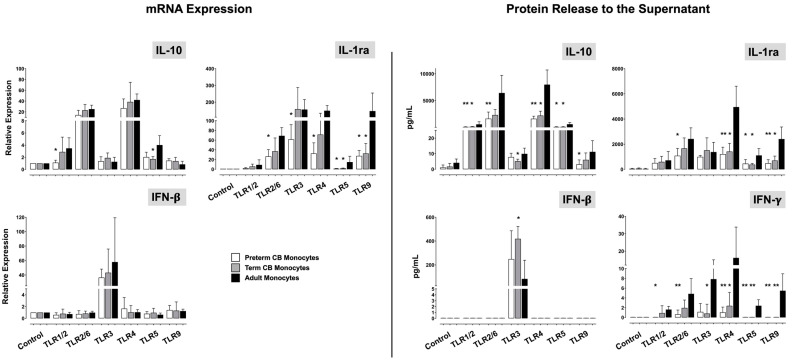
Anti-inflammatory IL-10 and IL-1ra, and IFN responses in TLR-stimulated preterm CB (white bars), term CB (grey), and adult monocytes (black). Unstimulated monocytes served as controls (baseline expression). Immune responses were assessed at the transcriptional and the translational level using qPCR and bead-based multiplex assay (mean ± SD; * *p* < 0.05, ** *p* < 0.01, vs. adult monocytes). For IFN-γ response, protein data are not accompanied by mRNA data. Contrary to the other mediators assessed in this study, IFN-γ mRNA was weakly expressed at 4 h incubation time throughout the study groups and CT values >30 did not allow for valid quantification.

Analyzing compensatory anti-inflammatory and immunoregulatory responses, we found lower induction of IL-10 and IL-1ra mRNA and, in particular, lower IL-10 and IL-1ra protein expression in stimulated preterm and term CB monocytes as compared with adults (Figure 2). Except for Pam3CSK4 and poly(I:C)-induced IL-1ra release, this phenomenon was independent of stimulus. As far as interferons were concerned, the poly(I:C)-driven release of IFN-β was higher in term CB monocytes compared with adults. For IFN-γ, on the contrary, preterm and term monocyte samples stood out with lower release as compared to adult controls—except for stimulation with poly(I:C), Pam3CSK4, and zymosan (Figure 2).

We determined the ratios of pro-inflammatory TNF-α, IL-1β, IL-6, and IL-8 protein to anti-inflammatory IL-10 and IL-1ra protein to better approximate the net effect of CB and adult monocyte responses upon individual TLR stimulation. Preterm and term CB monocytes showed higher ratios of pro-inflammatory to anti-inflammatory mediators as compared to adult controls for most stimuli (Table 2). Notably, the highest pro-inflammatory net effect was found in Pam3CSK4 (TLR1/2)-, zymosan (TLR2/6)-, and LPS (TLR4)-stimulated preterm and term CB samples, partly also upon exposure to the TLR3 and TLR5 ligand (Table 2).

### 2.4. Stimulus-Driven TLR Expression and TLR Signaling

To investigate whether differences in cytokine responses among the study groups were associated with differences in TLR expression and/or signaling, we assessed TLR mRNA expression and phosphorylation of the key signaling molecules p65, p38, ERK1/2, JNK, IRF3, and IRF7. Baseline expression of the given TLRs and signaling molecules did not differ. Upon stimulation, on the contrary, we found a differentially modulated *TLR* expression in preterm and term CB monocytes compared to adult controls, especially for *TLR1*, *TLR2*, and *TLR4*.

**Figure 3:**
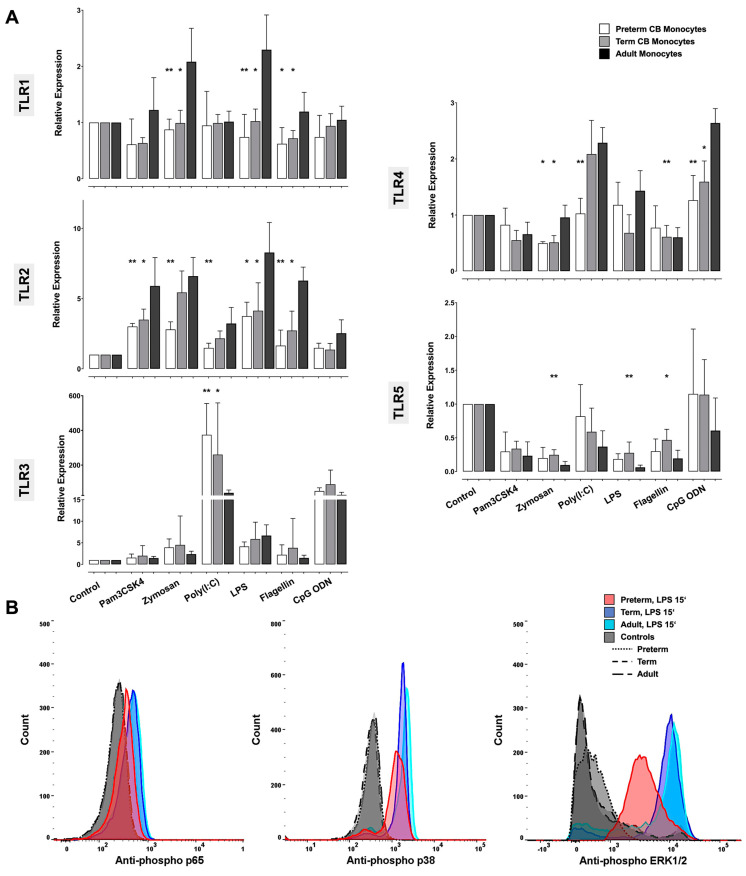
(**A**) Stimulus-induced mRNA expression of TLR1, 2, 3, 4, and 5 in preterm CB (white bars), term CB (grey), and adult monocytes (black) at baseline and upon Pam3CSK4, zymosan, poly(I:C), LPS, flagellin, and CpG ODN stimulation. Values represent the means ± SD (* *p* < 0.05, ** *p* < 0.01, vs. adult monocytes). For reason of chart readability, differences in expression levels within one group are presented in the text. (**B**) Stimulus-driven phosphorylation of p65, p38, and ERK1/2. Representative overlaid flow cytometer histograms show staining of phospho-p65, phospho-p38, and phospho-ERK1/2 in preterm CB (red), term CB (blue), and adult monocytes (turquoise) exposed to the TLR4 ligand LPS compared with unstimulated controls (grey; dashed and dotted lines).

*TLR1* expression was significantly lower in zymosan-, LPS-, and flagellin-stimulated CB monocytes compared with adults (Figure 3A) (increase by zymosan and LPS in adult monocytes, *p* < 0.05 vs. adult control). A similar pattern was detected for *TLR4*, with lower stimulus-induced expression in CB monocytes compared with adults (Figure 3A) (increase by poly(I:C) and CpG ODN in adult monocytes, *p* < 0.05 vs. adult control; decrease upon zymosan in preterm monocytes, *p* < 0.01 vs. preterm control). For *TLR2*, we saw an increase in all study groups compared with group-related baseline (Pam3CSK4: *p* < 0.001 vs. preterm, *p* < 0.01 vs. term, *p* < 0.05 vs. adult control. Zymosan: *p* < 0.01 vs. preterm, *p* < 0.0001 vs. term, *p* < 0.001 vs. adult control. LPS: *p* < 0.0001 vs. preterm, *p* < 0.01 vs. term, *p* < 0.0001 vs. adult control. Flagellin: *p* < 0.01 vs. adult control). However, this increase was lower in CB monocytes compared with adults (Figure 3A). *TLR3* increased significantly in all study groups upon stimulation with poly(I:C) (*p* < 0.0001 vs. preterm, *p* < 0.001 vs. term, *p* < 0.0001 vs. adult control) and CpG ODN (CpG ODN: *p* < 0.01 vs. preterm, *p* < 0.01 vs. term, *p* < 0.001 vs. adult control). This poly(I:C)-driven increase was higher in CB monocytes compared with adults (Figure 3A). *TLR5* was the only TLR analyzed whose mRNA expression levels decreased compared to group-related baseline in CB and in adult monocytes upon exposure to most ligands (Pam3CSK4: *p* < 0.05 vs. preterm, *p* < 0.05 vs. adult control. Zymosan: *p* < 0.01 vs. preterm, *p* < 0.01 vs. term, *p* < 0.001 vs. adult control. LPS: *p* < 0.01 vs. preterm, *p* < 0.01 vs. term, *p* < 0.0001 vs. adult control. Flagellin: *p* < 0.01 vs. adult control). This reduction was less pronounced in CB samples (Figure 3A).

Phosphorylation of p65, p38, and ERK1/2 in preterm and term CB monocytes paralleled phosphorylation in adults as assessed by flow cytometry. Pam3CSK4, zymosan, LPS, and flagellin induced a time-dependent phosphorylation of p65, p38, and ERK1/2 in all study groups (Figure 3B, Suppl. Table S1). Except for lower levels of LPS-driven ERK1/2 phosphorylation in preterm CB monocytes at 30 min incubation time, kinetics and levels of phosphorylation did not differ among the study groups (Suppl. Table S1). Ligand-induced p65 phosphorylation peaked at 15–30 min (Suppl. Table S1) and was only weakly detectable at 60 min (mean MFI ± SD upon LPS stimulation: preterm 111.3 ± 27.9; term 113.0 ± 28.8; adult 116.5 ± 55.8), while phosphorylation of p38 peaked at 15–30 min (Suppl. Table S1) and was detectable until 60 min (mean MFI ± SD upon LPS stimulation: preterm 347.3 ± 76.6; term 323.0 ± 130.1; adult 364.5 ± 55.2). Maximum phosphorylation of ERK1/2 was seen at 30 min (Suppl. Table S1) until 90 min (mean MFI ± SD upon LPS stimulation: preterm 10227.0 ± 3612.7; term 9456.1 ± 1636.4; adult 9233.3 ± 4142.8). Neither poly(I:C), a synthetic mimetic of viral double-stranded RNA, nor CpG ODN, a mimetic of oligonucleotide sequences common in bacterial and viral DNA, induced phosphorylation of p65, p38, and ERK1/2. Phosphorylation of JNK kinase, IRF3, and IRF7 could not be determined in any of the groups at any of the chosen time points.

### 2.5. Differential Modulation of Subset Frequencies in Stimulated Cord Blood Monocytes

**Figure 4:**
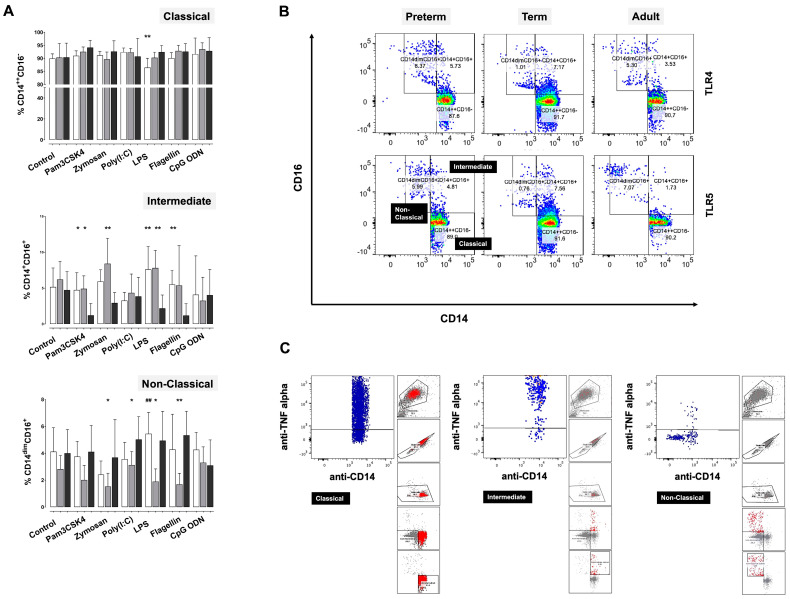
(**A**) Frequencies of CD14^++^CD16^−^ (classical), CD14^+^CD16^+^ (intermediate), and CD14^dim^CD16^+^ (non-classical) monocyte subsets at baseline and in TLR-stimulated preterm CB (white bars), term CB (grey), and adult monocytes (black) as assessed by flow cytometry (* *p* < 0.05; ** *p* < 0.01 vs. adult; ## *p* < 0.01 vs. term CB). (**B**) Representative dot plots of monocyte subpopulations upon stimulation with LPS (TLR4 ligand) and flagellin (TLR5). (**C**) Representative dot plots of TNF-α/surface marker co-staining in LPS-stimulated preterm CB monocytes. The ancestry panels of each dot plot display the backgating of each subset as well as the gating strategy applied. Monocytes were identified via forward and side scatter. Doublets and dead cells were excluded, as was the population of “not CD14^+^CD16^+^ monocytes”. Frequencies of classical (CD14^++^CD16^−^), intermediate (CD14^+^CD16^+^), and non-classical (CD14^dim^CD16^+^) monocytes were determined using rectangular gating.

Given the scarcity of data on monocyte heterogeneity in neonates, we examined the frequencies and functional responses of monocyte subpopulations in preterm and term CB versus adult monocyte samples. At baseline, percentages of classical (CD14^++^CD16^−^), intermediate (CD14^+^CD16^+^), and non-classical monocytes (CD14^dim^CD16^+^) did not differ among the study groups (Figure 4A). Upon TLR stimulation, frequencies were differentially modulated in preterm and term CB monocytes—in particular, those of the intermediate and non-classical subset (Figure 4A). Percentages of intermediate monocytes in preterm and term samples were higher than in adults following exposure to Pam3CSK4, zymosan, LPS, and flagellin (Figure 4A,B). For non-classical monocytes, percentages were lower in zymosan-, poly(I:C)-, LPS-, and flagellin-stimulated newborn CB. In preterm CB monocytes, frequencies of the non-classical subset showed the same tendency upon stimulation with zymosan and poly(I:C), but differences did not reach statistical significance (Figure 4A,B). Frequencies of classical monocytes differed only in LPS-stimulated samples with preterm CB monocytes presenting with lower frequencies as compared to adult controls (Figure 4A).

Using parallel staining for intracellularly accumulated cytokines, we assessed inflammatory capacities and tested for a relationship with an individual subset with distinct cytokine responses. Independent of stimulus, we identified classical and intermediate monocytes as the main source of pro-inflammatory TNF-α and IL-1β (TNF-α release exemplary in LPS stimulated monocytes: classical subset: preterm CB 43.3 ± 9.4% positive cells, term CB 46.4 ± 11.0, adult monocytes 39.0 ± 2.8; intermediate subset: preterm CB 45.2 ± 7.5% positive cells, term CB 35.9 ± 8.0, adult monocytes 42.2 ± 13.3; non-classical subset: preterm CB 6.4 ± 5.0% positive cells, term CB 4.6 ± 3.8, adult monocytes 6.0 ± 2.9) (Figure 4C). The subset of non-classical monocytes was also positive for TNF-α and IL-1β, but numbers of positive cells were significantly lower than in classical and intermediate monocytes throughout all experiments (preterm CB, term CB, and adult monocytes, *p* < 0.05 and *p* < 0.01 vs. classical and intermediate monocytes) (Figure 4C). Anti-inflammatory IL-10, on the contrary, was predominantly detected in classical monocytes (exemplary for LPS stimulation: classical subset: preterm CB 18.5 ± 6.2% positive cells, term CB 22.3 ± 4.3, adult monocytes 18.2 ± 1.6; intermediate subset: preterm CB 3.3 ± 1.0% positive cells, term CB 3.6 ± 1.5, adult monocytes 2.6 ± 1.1; non-classical subset: preterm CB 0.6 ± 0.1% positive cells, term CB 0.3 ± 0.3, adult monocytes 0.6 ± 0.5). IFN-β was spotted in classical and intermediate monocytes upon poly(I:C) stimulation. Functional characteristics did not differ among the study groups.

## 3. Discussion

This comprehensive evaluation of functional capacities of preterm and term CB monocytes upon stimulation with an array of well-known TLR ligands revealed that pro-inflammatory immune responses of term CB monocytes equal adult controls independent of stimulus. Except for IL-1β release, these findings also held true for preterm CB monocytes. In contrast, preterm and term CB monocytes released lower amounts of anti-inflammatory IL-10 and IL-1ra and immunoregulatory IFN-γ and stood out with higher ratios of pro-inflammatory to anti-inflammatory cytokines. Notably, we did not find major differences in the quality and quantity of immune responses upon individual TLR activation comparing CB and adult monocytes. Moreover, kinetics of p65, p38, and ERK1/2 phosphorylation equaled adult controls.

Our data do not support the hypothesis of globally impaired immune responses in moderate preterm and term CB monocytes, but rather match the clinical observation that this patient population frequently presents with a severe systemic inflammatory response during sepsis [33,34]. Moreover, present findings of robust pro-inflammatory responses are in line with cytokine measurements in plasma samples of preterm and term infants with severe infections [35,36,37]. In contrast, the present study indicates that the anti-inflammatory response is diminished and immature. These findings are in accordance with previous studies in term CB mononuclear cells showing diminished IL-10 production upon stimulation [25,32,38,39,40,41], and in preterm neonatal macrophages and whole blood displaying diminished IL-10 secretion upon LPS exposure [42,43]. Results of impaired IL-10 expression are also in line with cord blood cytokine levels assessed in term newborns compared with adult controls and in preterm infants with and without *Ureaplasma* infection, as well as measurements of cytokine panels in bronchoalveolar lavage samples of preterm infants with later development of BPD [44,45,46]. Given that IL-10 and IL-1ra physiologically control inflammation and promote the maintenance of cytokine homeostasis [47], these data may indicate an inability to balance cytokine responses and a propensity towards an inflammatory state. This feature may bear the risk of exaggerated inflammation and might account for the development of inflammation-related disorders and increased infection-related morbidity and mortality [4]. There is substantial evidence that inadequate anti-inflammatory responses may have detrimental consequences in the event of infection and inflammation, correlating with greater sepsis severity in neonates and predisposing them to neonatal lung injury [4,44,48]. Underlying mechanisms need to be further investigated. So far, the impaired resolution of inflammation has been described due to reduced neonatal phagocyte function as well as altered apoptosis and prolonged survival of neonatal mononuclear cells [3,49,50]. Moreover, adenosine, cAMP, and S100 alarmin levels have been discussed as factors modulating the neonatal innate immune response [23,51,52]. A recent study in preterm and term monocyte-derived macrophages suggested a differential modulation of major transcriptional regulators as an underlying mechanism [9]. Finally, epigenetics and post-transcriptional regulation have been acknowledged as potential contributors to neonatal-specific responses [53,54,55]. In this context, it is worth discussing that we found a discrepancy between IL-10 mRNA and protein levels among CB and adult monocytes, with major differences at the translational but not transcriptional level, especially upon stimulation with Pam3CSK, zymosan, and LPS. These findings might point to differences in the post-transcriptional control of anti-inflammatory cytokines and IL-10, in particular in preterm and term monocytes as reported, e.g., in autoimmune diseases [47,56,57]. Other mechanisms described for *IL10* gene regulation include epigenetic control, activation of individual transcription factors, or activation of specific intracellular signaling cascades [58]. Notably, different stability profiles were found for IL-10 mRNA expression in mouse macrophages upon stimulation with Pam3CSK4 and subsequent TLR2 signaling versus stimulation with LPS and consecutive TLR4 signaling [57]. Enhanced stability of IL-10 mRNA upon activation of TLR4 and Toll/IL-1 receptor domain-containing adaptor inducing IFN-β (TRIF) has been discussed as an underlying mechanism [57]. It is worth acknowledging that we found a differential modulation of TLR expression in stimulated CB monocytes compared to adults—in particular, concerning *TLR1, TLR2*, and *TLR4* expression. These findings are in keeping with previous studies in neonatal peripheral blood mononuclear cells, monocytes, and granulocytes [9,59,60]. In summary, differences in the post-transcriptional regulation of IL-10 expression, either caused or accompanied by a stimulus-driven modulation of TLR expression in neonatal monocytes, might impact the pattern of immune response.

Our study is one of few investigations so far to examine frequencies of monocyte subsets in preterm CB monocytes [20]. In accordance with a previous analysis in newborn CB samples [61], baseline frequencies in preterm and term CB monocytes did not differ from adult controls. Upon TLR stimulation, however, intermediate monocytes (CD14^+^CD16^+^) were more abundant both in stimulated preterm and term CB samples as compared to adults. These results are mostly in line with few existing studies in preterm and term neonates and young children below two years of age. Higher frequencies of intermediate monocytes were reported at baseline [62,63,64] and in in utero or ex vivo stimulated CB monocytes [61,65]. A significant expansion of the intermediate subset was found in preterm and term neonates and small children with sepsis [66,67]. In the present study, classical and intermediate monocytes were the main source of pro-inflammatory cytokines. IL-10 was mainly detected in the classical subset. In adults, intermediate monocytes have been described as the main source of pro-inflammatory IL-6 and IL-8 and as a key subset expanded in endotoxemia [68]. Given these data, one may speculate that higher frequencies of intermediate monocytes in preterm and term CB may further promote an inflammatory state. This assumption requires further research. Remarkably, in our study, the expansion of the intermediate subset in CB samples was observed mainly upon stimulation with Pam3CSK4 (TLR1/2), zymosan (TR2/6), and LPS (TLR4). At the same time, TLR1/2-, TLR2/6-, and TLR4-stimulated preterm and term CB monocytes stood out with the highest pro-inflammatory net effect. Given that TLR2 and TLR4 provide an innate immune response against gram-positive and gram-negative bacteria [7,21], one might assume that CB monocytes are capable of mounting particularly potent pro-inflammatory responses against pathogens frequently involved in chorioamnionitis and neonatal sepsis [48,69]. Of note, in adult studies, intermediate monocytes seemed to express higher levels of TLR2, TLR4, and TLR5 as compared to the other subsets [13,14,16,70]. In preterm monocyte subsets, no differences in TLR2 expression but lower expression of TLR4 in classical monocytes were found [62]. In our study, we cannot rule out the possibility that differences in stimulus-driven modulation of TLR2 or TLR4 expression were associated with characteristics and frequencies of individual monocyte subsets since our staining strategy was limited to eight fluorescence channels provided by our flow cytometer.

While our results indicate that the pro-inflammatory responsiveness of both cell surface and intracellularly localized TLRs in moderate preterm and term CB monocytes matches that of adults, data from previous studies are partly conflicting. Findings of impaired pro-inflammatory responses in neonatal whole blood [8,26,41,71,72] and CB monocytes [32,72,73,74], as well as attenuated phosphorylation of p38, ERK1/2, and NF-κB [5,62,75], contrast with reports of comparable or even exaggerated inflammatory responses in neonatal whole blood [6,76], monocyte-derived macrophages and dendritic cells [9,32], and CB monocytes [24,25,61,77,78], and equal or higher phosphorylation of p38 and ERK1/2 [22,61]. Moreover, some investigations described generally impaired TNF-α production in stimulated preterm and term CB mononuclear cells [22,23,24,25], while others reported attenuated immune responses upon activation of TLR1/2, TLR2/6, TLR4, and TLR5 signaling but robust responses to ligands mimicking viral infection, such as the TLR3 ligand poly(I:C) [26,27,28,29]. However, others found that TLR1/2- and TLR2/6-driven immune responses equal those of adults while other TLR responses seem impaired [30,31,32]. Data disparities may be due, to some extent, to methodological differences including the mononuclear cell type used and the timing or technique of sampling. Cell type–specific immune responses have been described with partly significant differences between monocytes, dendritic cells, and macrophages [32,79,80]. Moreover, CB cytokine responses may differ from peripheral blood monocyte responses at 1–4 weeks of age [51,81], and cytokine levels may depend on incubation times [24,26,82]. In the present study, the latter were defined according to preliminary experiments aimed at the detection of peak levels of the chosen parameters. However, we cannot exclude the possibility that assessments at other time points would have generated different results. Of note, Levy et al. found impaired TNF-α responses in newborn whole blood compared to adults upon stimulation with ligands of TLR1/2, TLR2/6, and TLR4 at 3 h, 5 h, and 8 h incubation, whereas levels of TNF-α release at 24 h did not differ [26]. Moreover, this study detected lower p38 phosphorylation upon LPS stimulation at 10 min, but equal levels in term CB and adults at 30 min [26]. Finally, it has to be acknowledged that monocyte responses in vivo might be distinct from the ex vivo setting since neither soluble modulators of TLR activation are present in assays with purified cells nor does this approach integrate the complex interaction of innate and adaptive immune cells, including antigen-presenting cells, subclasses of T lymphocytes, such as regulatory T cells and B cells, and the crosstalk with microbiota [4,22,23,26,51,61,83,84]. However, monocytes isolated from preterm and term cord blood samples provide a useful tool to study monocyte-driven innate immune function in the context of individual stimuli. Findings of differentially modulated monocyte subsets, as in the present study, contribute to the rapidly emerging field of research on monocyte heterogeneity.

However, our investigation did have some limitations. Firstly, it is a major weakness that we were not able to include additional phenotypic markers in our staining strategy [15,20,63]. Future studies will incorporate HLA-DR, CD36, CCR2, and CD11c, as well as TLR surface expression, to allow analysis of phenotype und function of neonatal monocyte subsets in more detail. Moreover, phagocytosis assays which are missing in the present study ought to further characterize functional capacities of monocyte subsets in our future research. Secondly, a notable limitation of this study is related to the small sample size and the lack of CB monocyte samples obtained from preterm infants at <30 weeks’ gestation. The findings obtained in the present study need to be confirmed in trials with lager sample sizes and, at best, ought to be correlated with in vivo cytokine analyses. Finally, it needs to be taken into account that monocyte selection techniques might impact phenotypic and functional analyses. Notably, comparing positive and negative selection, a previous study demonstrated that CD14 positive selection resulted in a lower percentage of intermediate monocytes compared with untouched peripheral blood mononuclear cells (PBMCs) but did not affect numbers of the classical and non-classical subset [85]. Negative selection, on the contrary, resulted in a significantly different distribution of subsets compared with untouched PBMCs, with a higher percentage of classical monocytes, and an absence of intermediate and non-classical monocytes [85], indicating that negative selection techniques might be disadvantageous in ex vivo studies analyzing monocyte subpopulations.

## 4. Materials and Methods

### 4.1. Study Cohort

CB samples of preterm (*n* = 8) and term neonates (*n* = 8) were obtained at the level III neonatal intensive care units at the University Children’s Hospital Würzburg and the University of Leipzig Medical Center between January 2019 and January 2021. Inclusion criteria for preterm infants were gestational age (GA) 32 0/7 to 36 6/7 weeks and delivery by C-section due to maternal preeclampsia or maternal hypertension. Inclusion criteria for term neonates were GA 37 0/7 to 41 6/7 weeks and delivery by elective C-section. Exclusion criteria comprised clinical or laboratory evidence of chorioamnionitis, suspected or confirmed perinatal infection, congenital malformation, or lack of written consent. GA was calculated based on early prenatal ultrasound. Placental histopathological examinations were performed at the Institute of Pathology, University of Würzburg. Adult monocytes were isolated from by-products of apheresis donations of healthy donors (>18 years; *n* = 8).

### 4.2. Sample Collection

CB samples were collected using a closed collection system (Maco Pharma International, Tourcouing, France). CB was collected by the attending obstetrician in the operating theater. Adult samples were provided by the Institute of Clinical Transfusion Medicine and Hemotherapy, University of Würzburg.

### 4.3. Isolation of Neonatal and Adult CD14^+^ Monocytes

Within 2 h of collection, CB and adult PBMCs were accumulated by Ficoll-Paque gradient centrifugation (LINARIS GmbH, Mannheim, Germany). Hereafter, CD14^+^ monocytes were isolated by magnetic-activated cell sorting using CD14 MicroBeads^®^ (Miltenyi Biotec, Bergisch Gladbach, Germany) according to the manufacturer’s protocol. CD14^+^ monocytes were re-suspended in RPMI 1640. CD14^+^ purity was proven >90% by flow cytometry. Preliminary experiments testing positively, and negatively selected monocytes confirmed that the population isolated using CD14 MicroBeads^®^ could be activated without limitations. Monocytes were transferred into 24-well culture plates without pooling and seeded at a density of 1 × 10^6^ cells/mL. Cells rested for 2 h.

### 4.4. TLR Ligands and Stimulation Assays

Monocytes were either left untreated or stimulated with 100 ng/mL Pam3CSK4 (synthetic bacterial lipopeptide, TLR1/2 ligand; Invivogen, San Diego, CA, USA), 1 µg/mL zymosan (cell wall from *Saccharomyces cerevisiae*, TLR2/6 ligand; Invivogen), 50 µg/mL poly(I:C) (synthetic analog of double-stranded RNA, TLR3; Invivogen), 100 ng/mL lipopolysaccharide (LPS, *Escherichia coli* 055:B5, TLR4; Sigma-Aldrich), 100 ng/mL flagellin (purified from *S. typhimurium*, TLR5; Invivogen), and 10 µg/mL CpG ODN (class C CpG oligonucleotide, TLR9; Invivogen). All ligands were diluted in sterile endotoxin-free water. Using the ToxinSensor™ Endotoxin Detection System (GenScript, Piscataway, NJ, USA), we confirmed endotoxin levels < 0.06 EU/mL in any of the ligands used. Both stimulated cells and unstimulated controls were incubated at 37 °C in a humidified atmosphere with 5% CO_2_. Assays for qPCR, flow cytometry, and Luminex^®^ analyses were performed in each donor, using monocytes sampled from parallel wells. Transfection of poly(I:C) was performed using 4 μg/mL Lipofectin^®^ transfection reagent (ThermoFisher Scientific, Waltham, MA, USA) according to the manufacturer’s instructions. Optimum concentrations of ligands and incubation times were determined in preliminary dose-response and kinetic assays. For most cytokines, 4 h incubation allowed detection of peak mRNA levels, 14 h for detection of peak levels of intracellularly accumulated cytokines, and 24 h for peak protein levels in the supernatant. Viability of monocytes was confirmed ≥95% after 4 h, 14 h, and 24 h in naïve and TLR-stimulated monocytes. Stimulation in the presence of brefeldin A (10 μg/mL, Sigma-Aldrich) allowed for intracellular cytokine flow cytometry.

### 4.5. RNA Extraction, Reverse Transcription (RT-) PCR and Real-Time Quantitative Polymerase Chain Reaction (qPCR)

For RNA extraction, monocytes sampled were harvested per well after 4h of stimulation and separated by centrifugation at 340× *g* for 5 min. Total RNA was isolated using the NucleoSpin^®^ RNA Kit (Macherey-Nagel, Düren, Germany) according to the manufacturer’s instructions, eluted in 60 μL nuclease-free water, and stored at −80 °C until reverse transcription. Following assessment of purity and concentration, 0.12 to 0.32 µg, 0.13 to 0.30 µg, and 0.17 to 0.30 µg of total RNA of preterm CB, term CB, and adult monocytes were reverse transcribed using the High-Capacity cDNA Reverse Transcription Kit (Applied Biosystems, ThermoFisher Scientific, Waltham, MA, USA). cDNA synthesis was terminated by heating at 70°C for 10min. First-strand cDNA was stored at −80 °C until further processing. For quantitative detection of *TNF*, *IL1B*, *IL8*, *CCL5*, *CXCL10*, *IL10*, *IL1RA*, *IFNB1*, *IFNG*, *TLR1, TLR2*, *TLR3*, *TLR4*, and *TLR5* mRNA, cDNA was diluted 1:10 in deionized, nuclease-free water and analyzed in duplicates of 25 μL using 12.5 μL iTaq™ Universal SYBR Green Supermix (Bio-Rad Laboratories, Inc., Hercules, CA, USA) (for primer sequences see Table 3). Analysis was performed using a 7500 Real-Time PCR System (Applied Biosystems, ThermoFisher Scientific) and a CFX96 Real-Time PCR Detection System (Bio-Rad, Munich, Germany). Amplification was normalized to *peptidyl prolyl isomerase A* (*PPIA*) and mean fold changes of mRNA were calculated by the ΔΔC_T_ method.

### 4.6. Flow Cytometry

For assessment of intracellularly accumulated cytokines, monocytes were harvested at 14 h and stained with a fixable viability dye (eBioscience, ThermoFisher Scientific, Waltham, MA, USA), Pacific Blue- and PE-conjugated CD14 and CD16 antibodies (BioLegend, San Diego, CA, USA). Cells were centrifuged, washed in phosphate buffered saline (PBS) containing 1% human serum (HS) and fixed (Fixation buffer, BioLegend). Permeabilization using ice-cold methanol was followed by staining with monoclonal antibodies to TNF-α (PerCP/Cy5.5), IL-1β (FITC), IFN-β (Alexa Fluor 647), and IL-10 (PE/Cy7) (all BioLegend). Monocytes were washed and re-suspended in PBS/1%HS. For analysis of p65, p38, ERK1/2, JNK, IRF3, and IRF7 phosphorylation, monocyte aliquots were processed after 15 min, 30 min, 60 min, 90 min, 6 h, 12 h, and 18 h and fixed immediately, washed with PBS/1% HS and permeabilized at −20 °C (True-Phos™ Perm Buffer, BioLegend). Hereafter, monocytes were stained with monoclonal antibodies to phospho-p65 (Alexa Fluor 488, Cell Signaling Technology, Danvers, MA, USA), phospho-p38 (Pacific Blue, BD Biosciences, San Jose, CA, USA), phospho-ERK1/2 (PE/Cy7, BioLegend), and phospho-SAPK/JNK (PE, Cell Signaling), as well as phospho-IRF3 (Alexa Fluor 647, Cell Signaling) and IRF7 (Alexa Fluor 488, BD Biosciences), for 30 min at room temperature. Monocytes were re-suspended in PBS/1%HS. Data were acquired using a BD FACSCanto II flow cytometer and analyzed by FACSDiva v6.1.3 and FlowJo software v10 (BD, Franklin Lakes, NJ, USA). Instrument set-up and single stain compensation for correction of spectral spillover were performed prior to data acquisition. A baseline fluorescence control and an isotype-matched negative control served as reference to set fluorescence thresholds. Monocytes were gated via forward and side scatter, and doublets and dead cells were excluded (Figure 4C). Phosphorylation of p65, p38, ERK1/2, JNK, IRF3, and IRF7 was assessed by calculating the mean fluorescence intensity (MFI) of histograms (stained MFI *minus* MFI of matched isotype control).

### 4.7. Bead-Based Multiplex Assay of Secreted Cytokines

For quantification of released cytokines, supernatants were collected at 24 h and stored at −80 °C until analysis. Samples were evaluated for concentrations of TNF-α, IL-1β, IL-6, IL-8, IL-12p40, IL-10, IL-1ra, IFN-β, IFN-γ, CXCL10, and CCL5 using a customized Luminex^®^ multiplex cytokine/chemokine panel (Merck Millipore, Merck group, Darmstadt, Germany) according to the manufacturer’s instructions. Undiluted supernatants were measured in duplicate. Lower limits of detection were obtained from standard curve calculations for each analyte: 1.67pg/mL (TNF-α), 1.53 pg/mL (IL-1β), 3.75 pg/mL (IL-6), 1.51 pg/mL (IL-8), 2.31 pg/mL (IL-12p40), 0.88 pg/mL (IL-10), 2.56 pg/mL (IL-1ra), 7.83 pg/mL (IFN-β), 0.44 pg/mL (IFN-γ), 2.21 pg/mL (CXCL10), and 3.21 pg/mL (CCL5). Analyses were performed using xPONENT^®^ software (Luminex Cooperation, Austin, TX, USA).

### 4.8. Statistics

Prism^®^ 9 software (GraphPad Software, San Diego, CA, USA) was used for statistical analyses. Data are expressed as mean ± standard deviation (SD). The Kruskal-Wallis non-parametric test followed by Dunn’s multiple comparison post-hoc test was performed, comparing expression levels among the study groups and among different conditions within one group. Statistical significance was set at *p* < 0.05 for all analyses.

## 5. Conclusions

Our data confirm potent pro-inflammatory immune responses in preterm and term CB monocytes, but show diminished anti-inflammatory responses, independent of stimulus. CB monocytes seem equipped to initiate a protective pro-inflammatory response, while balancing cytokines might be impaired, bearing the risk of exaggerated or sustained inflammation. Notably, TLR1/2-, TLR2/6-, and TLR4-stimulated CB monocytes stood out with the highest pro-inflammatory net effect. Whether or not a differential expansion of intermediate monocytes promotes this inflammatory state needs to be further elucidated. In addition, findings of differentially modulated TLR expression but equal kinetics of p65, p38, and ERK1/2 phosphorylation ought to prompt further studies of TLR sensor function in neonatal monocytes.

## Figures and Tables

**Table 1:** Baseline characteristics of preterm and term cord blood donors.

Characteristics	Preterm Infants	Term Infants
	(*n* = 8)	(*n* = 8)
Gestational age (weeks), mean (range)	33 5/7 (32 3/7–35 0/7)	38 3/7 (37 3/7–39 1/7)
Birth weight (g), mean (range)	1960 (1410–2520)	3410 (2720–4100)
Sex (male), *n* (%)	5 (62.5%)	3 (37.5%)
Singleton, *n* (%)	8 (100%)	8 (100%)
Rupture of membranes > 2 h, *n* (%)	0 (0%)	0 (0%)
Histologic chorioamnionitis, *n* (%)	0 (0%)	0 (0%)
Caesarean section, *n* (%)	8 (100%)	8 (100%)

**Table 2:** Ratios of pro-inflammatory to anti-inflammatory cytokines.

			Control	Pam3 CSK4	Zymosan	Poly (I:C)	LPS	Flagellin	CpG ODN
TNF-α/IL-10	Preterm	Mean	2.44	6.82	6.80 **	6.61	3.50 *	5.47	74.34
protein		±SD	±5.02	±6.11	±4.79	±7.38	±2.56	±3.12	±111.25
	Term	Mean	0.24	10.76	3.82 *	6.02	3.46 *	6.08	2.68
		±SD	±0.42	±8.45	±2.08	±3.87	±1.95	±5.12	±1.90
	Adult	Mean	0.94	3.31	1.14	4.0	0.71	1.76	5.55
		±SD	±0.92	±2.25	±0.87	±3.16	±0.30	±0.96	±3.05
IL-1β/IL-10	Preterm	Mean	0.02	0.07	0.68	0.55	0.39	0.08	3.16
protein		±SD	±0.04	±0.03	±0.48	±0.80	±0.26	±0.04	±7.71
	Term	Mean	0	0.1	0.59	0.51	0.56	0.14	0.07
		±SD	±0	±0.05	±0.57	±0.45	±0.48	±0.08	±0.15
	Adult	Mean	0	0.08	0.45	0.2	0.35	0.07	0.10
		±SD	±0	±0.11	±0.70	±0.12	±0.28	±0.08	±0.14
IL-6/IL-10	Preterm	Mean	2.20	31.06 *	6.63 **	4.13	5.80 **	52.59 **	17.07
protein		±SD	±4.92	±27.93	±5.10	±6.33	±2.47	±38.31	±36.12
	Term	Mean	0.02	42.60 **	3.85 *	3.97	4.31 *	29.28	1.37
		±SD	±0.04	±41.15	±1.23	±2.92	±1.71	±15.24	±1.37
	Adult	Mean	0.41	6.13	0.84	1.75	0.60	7.44	3.50
		±SD	±0.56	±3.90	±0.97	±1.27	±0.45	±6.15	±1.80
IL-8/IL-10	Preterm	Mean	461.69	95.20 *	6.74 *	27.48	3.52	101.73 *	614.31
protein		±SD	±540.87	±35.81	±3.38	±21.45	±1.81	±62.73	±894.21
	Term	Mean	53.66	94.04 *	6.27	47.48 *	5.64 **	135.71 **	70.15
		±SD	±16,62	±30.26	±3.60	±16.65	±3.36	±75.11	±44.52
	Adult	Mean	29.63	17.34	2.2	16.96	1.37	17.80	78.26
		±SD	±20.58	±11.84	±2.37	±9.57	±1.04	±18.80	±47.81
TNF-α/IL-1ra	Preterm	Mean	0.03	1.70	8.84 **	0.04	4.13	1.27	0.05
protein		±SD	±0.05	±1.74	±4.46	±0.02	±1.64	±0.82	±0.03
	Term	Mean	0.02	2.26	5.96 *	0.02	5.25 **	1.4	0.04
		±SD	±0.40	±1.45	±4.40	±0.01	±2,40	±0.95	±0.02
	Adult	Mean	0.12	2.73	2.59	0.03	1.15	1.1	0.03
		±SD	±0.14	±1.76	±1.32	±0.01	±0.48	±1.1	±0.014
IL-1β/IL-1ra	Preterm	Mean	0.002	0.02	0.89	0.003	0.57	0.02	0.02
protein		±SD	±0.004	±0.01	±0.45	±0.005	±0.44	±0.02	±0.04
	Term	Mean	0	0.2	0.81	0.002	0.91	0.04	0.001
		±SD	±0	±0.01	±0.68	±0.004	±0.91	±0.03	±0.002
	Adult	Mean	0	0.07	0.88	0.002	0.45	0.03	0.002
		±SD	±0	±0.11	±1.17	±0.004	±0.12	±0.03	±0.002
IL-6/IL-1ra	Preterm	Mean	0	8.44	9.72 *	0.02	9.27 **	10.31	0.02
protein		±SD	±0	±11.76	±6.11	±0.02	±7.08	±4.04	±0.01
	Term	Mean	0	10.70	5.99	0.02	7.09 *	8.44	0.02
		±SD	±0	±12.99	±3.23	±0.01	±3.93	±5.18	±0.03
	Adult	Mean	0.06	5.41	1.82	0.01	0.97	4.67	0.03
		±SD	±0.12	±3.72	±1.61	±0.01	±0.85	±5.94	±0.05
IL-8/IL-1ra	Preterm	Mean	4.17	22.60	11.52	0.17	4.52	27.29	1.26
protein		±SD	±3.54	±14.47	±9.51	±0.06	±1.68	±22.55	±1.67
	Term	Mean	3.28	22.73	9.61	0.21	8.51 **	33.59 *	1.02
		±SD	±1.70	±9.29	±7.45	±0.09	±3.33	±6.67	±0.79
	Adult	Mean	4.73	15.54	5.02	0.16	2.30	8.47	0.70
		±SD	±6.08	±11.51	±4.28	±0.13	±2.08	±6.92	±1.23

* *p* < 0.05, ** *p* < 0.01, vs. adult monocytes.

**Table 3:** List of primer sequences used for RT-qPCR.

Gene Symbol	Sequence Accession No.	Forward Primer	Reverse Primer
*CCL5*	NM_002985	GCTGTCATCCTCATTGCTACTG	CTTGACCTGTGGACGACTG
*CXCL10*	NM_001565	AGCACCATGAATCAAACTG	TGTAGCAATGATCTCAACAC
*IL1B*	NM_000576	TTCATTGCTCAAGTGTCTG	GCACTTCATCTGTTTAGGG
*IL8*	NM_000584	CAGTGCATAAAGACATACTCC	TTTATGAATTCTCAGCCCTC
*IL10*	NM_000572	GCTGTCATCGATTTCTTCC	GTCAAACTCACTCATGGCT
*IL1RA*	NM_173842	CTTCTATCTGAGGAACAACCA	AGTGATGTTAACTGCCTCC
*IFNB1*	NM_002176	CTCTCCTGTTGTGCTTCTCC	TGTCAAAGTTCATCCTGTCCT
*IFNG*	NM_000619	TGGGTTCTCTTGGCTGTTA	CTGTCACTCTCCTCTTTCC
*PPIA*	NM_021130	CAGGGTTTATGTGTCAGGG	CCATCCAACCACTCAGTC
*TLR1*	NM_003263	GACTGCCAAATGGAACAGAC	TTAGTGTTCATGAAGACCCTG
*TLR2*	NM_003264	CCAAAGGAGACCTATAGTGAC	GCTTCAACCCACAACTACC
*TLR3*	NM_003265	GAGTGCCGTCTATTTGCC	TCTGTCTCATGATTCTGTTGG
*TLR4*	NM_138554	TTATCCAGGTGTGAAATCCA	GATTTGTCTCCACAGCCA
*TLR5*	NM_003268	GTCCTTTCTCCTGATTCACCA	GTCTCCCATGATCCTCGT
*TNF*	NM_000594	CAGCCTCTTCTCCTTCCT	GGGTTTGCTACAACATGG

## Data Availability

The datasets generated and analyzed in the present study are available from the corresponding author upon reasonable request.

## Supplementary Materials

The following supporting information can be downloaded at: https://www.mdpi.com/article/10.3390/ijms24054919/s1.
