# Supplementary material for: Imbalanced Inflammatory Responses in Preterm and Term Cord Blood Monocytes and Expansion of the CD14+CD16+ Subset upon Toll-like Receptor Stimulation
Source: Int J Mol Sci. 2023 Mar 3;24(5):4919. doi: 10.3390/ijms24054919 (PMC10002861; doi:10.3390/ijms24054919)
Supplement: Supplementary file 1 [file ijms-24-04919-s001.zip › ijms-2193894-supplementary.pdf]

Supplemental Table S1: **Phosphorylation of p65, p38 and ERK1/2 in preterm and term CB monocytes and adult monocytes upon stimulation with Pam3CSK4, zymosan, LPS and flagellin**

				Pam3CSK4	Zymosan	LPS	Flagellin
**phospho-p65**	**15min**	**Preterm**	Mean MFI	261.7	273.3	299.5	195
			± SD	± 48.0	± 54.9	± 124.9	± 14.8
		**Term**	Mean MFI	212.7	261	418.3	202
			± SD	± 28.1	± 57.6	± 129.0	± 44.2
		**Adult**	Mean MFI	243	371.3	415.8	189.7
			± SD	± 68.5	± 129.4	± 96.0	± 34.2
	**30min**	**Preterm**	Mean MFI	178	198.3	215.4	171.7
			± SD	± 71.8	± 97.1	± 76.5	± 35.1
		**Term**	Mean MFI	125.7	237.3	226.6	168
			± SD	± 44	± 102.8	± 56.2	± 44.1
		**Adult**	Mean MFI	162.7	138.7	209.4	165
			± SD	± 62.7	± 52.5	± 36.6	± 31.6
**phospho-p38**	**15min**	**Preterm**	Mean MFI	415.3	522.3	970.5	**301.7†**
			± SD	± 103.1	± 140.9	± 330.4	± 146.3
		**Term**	Mean MFI	428.3	440	1011.8	**282.7‡**
			± SD	± 144.9	± 230.7	± 274.3	± 87.8
		**Adult**	Mean MFI	549	590	1041	301
			± SD	± 187.1	± 351.9	± 359.1	± 96.2
	**30min**	**Preterm**	Mean MFI	333.7	280.7	496.2	290.7
			± SD	± 199.8	± 153.2	± 272.0	± 89.8
		**Term**	Mean MFI	206.7	227.3	400.2	134.7
			± SD	± 171.9	± 90.9	± 199.2	± 51.6
		**Adult**	Mean MFI	370.3	195.7	414,8	168.7
			± SD	± 43.7	± 43.9	± 127,7	± 39.4
**phospho-ERK1/2**	**15min**	**Preterm**	Mean MFI	2233.3	2056.7	2022.5	971.7
			± SD	± 814.5	± 767.2	± 1120.2	± 119
		**Term**	Mean MFI	876.7	1443.3	2740.3	**593.3***
			± SD	± 385.5	± 825.5	± 1786.0	± 283.6
		**Adult**	Mean MFI	1933.3	2466.7	3266.7	2233.3
			± SD	± 404.1	± 602.8	± 1069.3	± 550.8
	**30min**	**Preterm**	Mean MFI	4600	3466.7	**4750***	2766.7
			± SD	± 1946.8	± 1266.2	± 2493.3	± 1069.3
		**Term**	Mean MFI	4166.7	5270	10366.7	**2640‡**
			± SD	± 1795.4	± 2605.7	± 2025.7	± 1359.3
		**Adult**	Mean MFI	5300	6800	11050	**3166.7#**
			± SD	± 1153.3	± 3360.1	± 2260.5	± 1747.4

* p<0.05, vs. adult monocytes;
† p<0.05 vs. TLR4-stimulated preterm CB monocytes; ‡ p < 0.05 vs. TLR4-stimulated term CB monocytes;
# p<0.05 vs. TLR4-stimulated adult monocytes.
